# Gastrointestinal Bleeding as an Unusual Presentation of Groove Pancreatitis Complicated by Intramural Duodenal Hematoma

**DOI:** 10.5152/tjg.2026.26084

**Published:** 2026-04-17

**Authors:** Tugba Tolu, Yasemin Kaldirim Armutcuoglu, Tuba Yilmaz Yildirim, Muhsinem Yılmaz Duz, Hale Nur Tiras, Yesim Ozen Alahdab, Osman Cavit Ozdogan, Coskun Ozer Demirtas

**Affiliations:** 1 Department of Gastroenterology, Marmara University Faculty of Medicine, İstanbul, Türkiye; 2 Department of Internal Medicine, Marmara University Faculty of Medicine, İstanbul, Türkiye

To the Editor,

Groove pancreatitis (GP), also known as paraduodenal pancreatitis, is an uncommon form of chronic pancreatitis characterized by fibroinflammatory changes in the pancreaticoduodenal groove that may mimic pancreatic head carcinoma. Hemorrhagic complications are rare, and intramural duodenal hematoma (IDH) has been reported only in isolated cases associated with pancreatitis.^1^ We present a case of GP complicated by IDH manifesting as overt upper gastrointestinal bleeding. Written informed consent for publication of the clinical details and imaging findings was obtained from the patient.

A 47-year-old man with alcohol-related liver cirrhosis (Child–Pugh class: B, MELD-Na score: 14) and recurrent acute pancreatitis presented with severe epigastric, belt-like abdominal pain, nausea, and coffee-ground vomiting. The patient had no history of smoking. He also reported intermittent melena during the preceding year. During the preceding 2 months, he was hospitalized on 4 occasions for alcohol-related acute pancreatitis.

During prior admissions, cross-sectional imaging demonstrated peripancreatic inflammatory changes and small cystic lesions. Magnetic resonance imaging performed on July 4 demonstrated inflammatory changes localized to the pancreaticoduodenal groove, with T2-weighted hyperintensity between the pancreatic head and the medial duodenal wall, consistent with segmental GP. The main pancreatic duct was mildly dilated (5 mm), and a previously described cystic lesion was reinterpreted as arising from the groove rather than the pancreatic parenchyma. On the current admission (August 23), the patient was hemodynamically stable. Laboratory evaluation revealed elevation of pancreatic enzymes (amylase: 1635 U/L, lipase: 2898 U/L), transaminase elevation (aspartate aminotransferase: 506 U/L, alanine aminotransferase: 289 U/L), and hyperbilirubinemia (total bilirubin: 2.46 mg/dL). Hemoglobin decreased from 16.6 g/dL at presentation to 12.0 g/dL during the hospital course. Platelet count was mildly reduced (119–150 × 10^9^/L); serum albumin was 3.4 g/dL and INR was 1.29. Contrast-enhanced computed tomography performed on August 25 demonstrated a 9 × 6 cm heterogeneous hyperdense lesion centered within the pancreaticoduodenal groove and extending into the medial wall of the second portion of the duodenum. The lesion showed heterogeneous hyperattenuation without internal enhancement, findings compatible with acute intramural hemorrhage. The main pancreatic duct remained mildly dilated, similar to prior imaging (Figure 1). Minimal perihepatic and pelvic free fluid was noted, with insufficient volume for diagnostic paracentesis.

Upper gastrointestinal endoscopy revealed a subepithelial bulge in the second portion of the duodenum causing significant luminal narrowing, with overlying mucosal edema and hyperemia. No active bleeding was observed during endoscopy (Figure 2). In the absence of active hemorrhage, no endoscopic hemostatic intervention or biopsy was performed. Esophageal and gastric varices were absent, and no alternative sources of gastrointestinal bleeding were identified. Intravenous proton pump inhibitor therapy was initiated due to suspected upper gastrointestinal bleeding. Computed tomography angiography demonstrated no contrast extravasation or arterial communication; therefore, no embolization or surgical intervention was pursued. The patient was managed conservatively with bowel rest, intravenous fluids, proton pump inhibitor therapy, and close clinical monitoring. Abdominal pain gradually improved, and hemoglobin levels remained unchanged at 12 g/dL over the subsequent 72 hours. No recurrent signs of gastrointestinal bleeding occurred. The patient was discharged in stable condition. Follow-up endoscopy 4 weeks later demonstrated complete resolution of the duodenal lesion. These findings were consistent with spontaneous resolution of the IDH. After discharge, the patient received counseling for alcohol abstinence and was scheduled for gastroenterology and hepatology follow-up.

Intramural duodenal hematoma is an uncommon condition most frequently associated with abdominal trauma, anticoagulant therapy, endoscopic procedures, or bleeding diatheses.^2^^,^^3^ Spontaneous, non-traumatic IDH is rare, and its occurrence in association with pancreatic disease is even more unusual. Reports describing IDH in the setting of paraduodenal pancreatitis are exceedingly limited, and the underlying pathophysiology remains incompletely understood.^3^

A bidirectional relationship appears to exist between pancreatitis and IDH. One proposed mechanism suggests that intramural hemorrhage may cause duodenal or periampullary obstruction, leading to impaired pancreatic ductal drainage and secondary pancreatitis. Conversely, pancreatic inflammation during acute exacerbations of chronic pancreatitis may result in enzymatic injury to adjacent duodenal submucosal vessels, subsequently causing intramural bleeding and hematoma formation.^2^^-^^5^ In the present case, prior imaging demonstrated groove-centered inflammation before the development of the hematoma, supporting pancreatitis as the initiating event.

Although the patient had underlying cirrhosis with mild thrombocytopenia, there was no evidence of significant coagulopathy, anticoagulant exposure, or traumatic trigger that would independently explain spontaneous hematoma formation. Taken together, these observations suggest that acute inflammatory exacerbation of GP was the more likely precipitating factor for vascular injury and secondary intramural bleeding. Hematoma-induced ductal obstruction as the primary event appears less probable in this clinical context. Such presentation should be considered when compatible imaging findings are present. Malignancy and other bleeding sources were excluded endoscopically.

Diagnosis of IDH relies primarily on cross-sectional imaging. Computed tomography typically demonstrates circumferential duodenal wall thickening with heterogeneous intramural attenuation and luminal narrowing, findings that may mimic malignancy. Endoscopy serves a complementary role; however, biopsy was deferred during the acute phase due to concern for re-bleeding.

Management is guided by hemodynamic status and the presence of active bleeding or associated complications. In hemodynamically stable patients without contrast extravasation, conservative treatment is generally appropriate. In contrast, cases complicated by ongoing hemorrhage or radiologic evidence of arterial communication have required endoscopic, radiologic, or surgical intervention.^2^^,^^3^^,^^6^ Notably, despite the presence of a large intramural hematoma and overt upper gastrointestinal bleeding, our patient remained clinically stable, with no contrast extravasation on angiography and no recurrent bleeding. Conservative management was therefore pursued, and invasive intervention was not required.

Beyond its rarity, this case underscores several clinically relevant points. First, it demonstrates that upper gastrointestinal bleeding may occur as an atypical presentation in the setting of GP. Although uncommon, such presentation should be considered in the differential diagnosis when compatible imaging findings are present. Second, a hyperdense mass-like lesion in the groove region extending into the duodenal wall may mimic malignancy; recognition of attenuation characteristics and lack of enhancement helps differentiate intramural hematoma from neoplastic disease. Third, despite the presence of a large hematoma and overt upper gastrointestinal bleeding, conservative management was feasible in a hemodynamically stable patient without radiologic evidence of active extravasation, underscoring the importance of individualized clinical assessment.

## Figures and Tables

**Figure 1.**
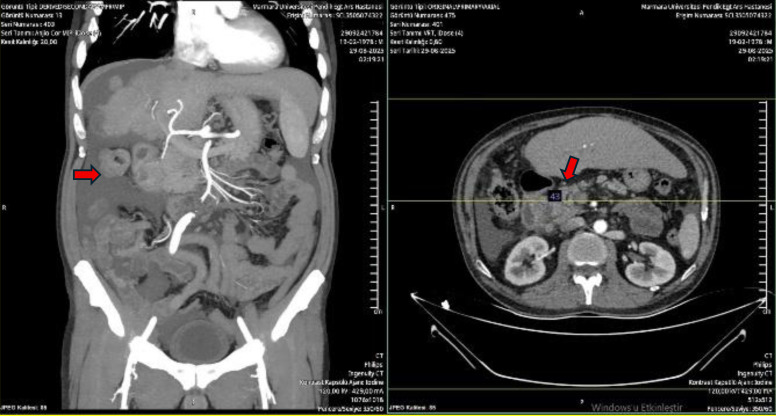
Coronal (left) and axial (right) contrast-enhanced CT images demonstrating a large heterogeneous hyperdense lesion centered in the pancreaticoduodenal groove and extending into the medial wall of the second portion of the duodenum. The lesion is associated with marked luminal narrowing, compatible with intramural duodenal hematoma.

**Figure 2.**
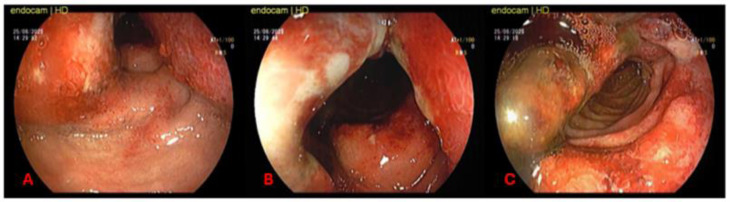
Endoscopic findings. (A) Edematous and hyperemic mucosa at the pyloric inlet. (B) Marked inflammatory narrowing at the pyloric channel. (C) Second portion of the duodenum demonstrating circumferential luminal narrowing due to intramural expansion consistent with hematoma, without endoscopic evidence of active bleeding.

## Data Availability

The data that support the findings of this study are available on request from the corresponding author.
